# Genetic cardiomyopathy unmasked by pregnancy: X-linked dystrophinopathy presenting as peripartum cardiomyopathy—a case report

**DOI:** 10.1093/ehjcr/ytag405

**Published:** 2026-06-02

**Authors:** Julian Bauer, Fabian Knebel, Wilhelm Haverkamp, Karin Klingel, Aleksandar Aleksandrov

**Affiliations:** Department of Internal Medicine II, Cardiology, Sana Klinikum Lichtenberg, Fanningerstraße 32, Berlin 10365, Germany; Department of Internal Medicine II, Cardiology, Sana Klinikum Lichtenberg, Fanningerstraße 32, Berlin 10365, Germany; Department of Cardiology, Angiology and Intensive Care Medicine, Charité Campus Mitte, German Heart Center of the Charité-University Medicine, Augustenburger Pl. 1, Berlin 13353, Germany; Cardiopathology, Institute for Pathology and Neuropathology, University Hospital Tübingen, Liebermeisterstraße 8, Tübingen 72076, Germany; Department of Internal Medicine II, Cardiology, Sana Klinikum Lichtenberg, Fanningerstraße 32, Berlin 10365, Germany

**Keywords:** Case report, Peripartum cardiomyopathy, Dystrophin gene, Genetic predisposition, Heart failure

## Abstract

**Background:**

Peripartum cardiomyopathy (PPCM) is an uncommon cause of heart failure occurring towards the end of pregnancy or in the months following delivery. Although often considered idiopathic, emerging evidence suggests a significant genetic contribution in some patients.

**Case summary:**

We report a 31-year-old woman who presented 10 weeks postpartum with symptoms of advanced heart failure. Transthoracic echocardiography revealed a severely reduced left ventricular ejection fraction (18%). Cardiac magnetic resonance imaging confirmed severe systolic dysfunction with non-ischaemic late gadolinium enhancement. After exclusion of other causes, PPCM was diagnosed. She was initiated on guideline-directed heart failure therapy and bromocriptine for lactation suppression. As part of the ongoing evaluation, endomyocardial biopsy revealed persistence of parvovirus B19 without histological evidence of active myocarditis. Genetic testing was initiated, and results later identified a pathogenic X-linked deletion of exons 44–50 in the dystrophin (DMD) gene, consistent with cardiac-restricted dystrophinopathy. The patient has remained clinically stable under ongoing therapy.

**Discussion:**

This case underscores the importance of considering genetic testing in PPCM. The identification of an X-linked dystrophin gene deletion was unexpected, as the patient had no neuromuscular symptoms. Such mutations may manifest as isolated cardiomyopathy in female carriers, with pregnancy serving as a precipitating physiological stressor. Identifying a genetic cause enables optimized management and facilitates targeted family screening.

Learning points

- Peripartum cardiomyopathy (PPCM) may present as the first clinical manifestation of an underlying genetic cardiomyopathy, even in the absence of a positive family history.
- Up to 15% of PPCM patients carry pathogenic cardiomyopathy-related variants, including pathogenic dystrophin variants, influencing management and familial risk stratification.
- Although genetic testing in PPCM patients is recommended as class IIb (‘may be considered’), this case shows the relevance of genetic testing, particularly in those with persistent left ventricular dysfunction or atypical imaging features.
- If available, consider genetic testing before myocardial biopsy; if a myocardial biopsy is performed, also perform dystrophin staining alongside electron microscopy.

## Introduction

Peripartum cardiomyopathy (PPCM) is a rare but potentially life-threatening condition, defined as the development of systolic heart failure (HF) during the peripartum period or in the months following delivery, termination, or miscarriage, with an incidence of 1 in 2000–5000 births.^1^ Risk factors include hypertensive pregnancy disorders, multiple gestation, advanced maternal age, prior PPCM, and African ancestry; despite partial recovery in many patients, mortality may reach 20%.^1–4^ Genetic susceptibility appears increasingly relevant in PPCM. Besides the well-recognized contribution of truncating variants in titin (TTN), several other cardiomyopathy-associated genes (e.g. SCN5A, BAG3, SYNM, and sarcomeric genes) have been implicated. Pathogenic dystrophin variants represent a less common mechanism by which impaired sarcolemmal stability may allow peripartum stressors to unmask X-linked cardiomyopathy.^4–6^ Although genetic testing was performed retrospectively, this case highlights the value of identifying a genetic aetiology at the index presentation. Earlier testing might have streamlined the diagnostic work-up, obviated invasive procedures, and enabled timely counselling and cascade screening, in accordance with current ESC recommendations.^7^

## Summary figure

**Figure:**
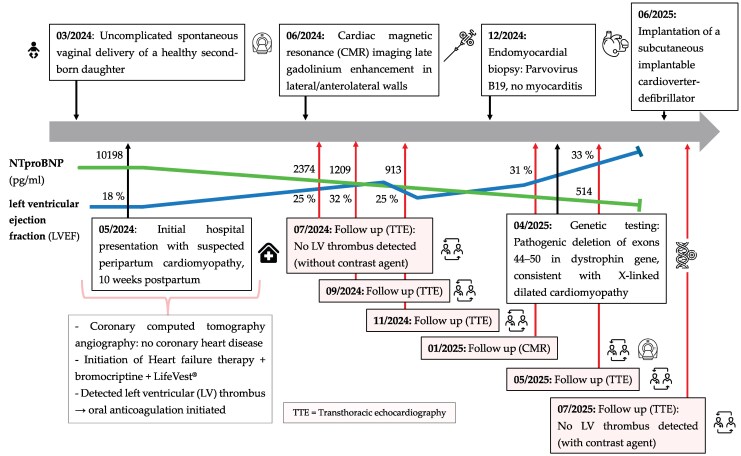


## Case presentation

A 32-year-old woman presented to our cardiology department in May 2024, 10 weeks postpartum, with a 5-day history of epigastric pain, nausea, vomiting, diarrhoea, exertional dyspnoea [New York Heart Association (NYHA) class II], and palpitations. She had delivered a healthy female infant via uncomplicated vaginal birth and was breastfeeding. Obstetric history included a prior uneventful term delivery of a healthy male infant in early 2023. She had no history of cardiovascular or neuromuscular disease, did not smoke, and took no regular medication other than intermittent paracetamol.

Physical examination was unremarkable. Outpatient laboratory tests showed elevated transaminases and bilirubin; abdominal ultrasound demonstrated gallbladder wall thickening. Transthoracic echocardiography in the emergency department revealed a severely reduced left ventricular ejection fraction (LVEF) of 18% (see Supplementary material online, *Video S1*), with moderate mitral regurgitation, severe tricuspid regurgitation, and grade III diastolic dysfunction (*Figure 1*). N-terminal pro B-type natriuretic peptide (NT-proBNP) was markedly elevated at 10 198 pg/mL, and high-sensitivity troponin I was 96 pg/mL. Electrocardiogram showed low peripheral voltage. The patient was admitted for acute HF management. Given the close temporal association with childbirth, these findings supported a working diagnosis of PPCM.

**Figure 1:**
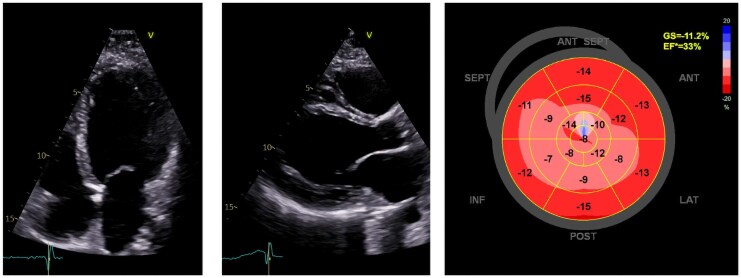
Echocardiographic findings showing a severely dilated left ventricle (end-diastolic volume 175 mL/96 mL/m^2^) with severely reduced global systolic function and marked global hypokinesia. The left ventricular ejection fraction, assessed biplane by Simpson’s method, is 18%. Global longitudinal strain is reduced to −11.1%.

Lactation was suppressed with bromocriptine to reduce cardiac workload. Guideline-directed medical therapy (GDMT) for HF with reduced ejection fraction was initiated, including sacubitril/valsartan, beta-blocker, sodium-glucose cotransporter-2 (SGLT-2) inhibitor, and spironolactone.

Contrast-enhanced echocardiography detected a left ventricular thrombus (*Figure 2*; Supplementary material online, *Videos S2* and *S3*), prompting initiation of oral anticoagulation at therapeutic dose. Coronary computed tomography angiography excluded significant coronary artery disease. The patient was discharged with a wearable cardioverter-defibrillator (LifeVest®).

**Figure 2:**
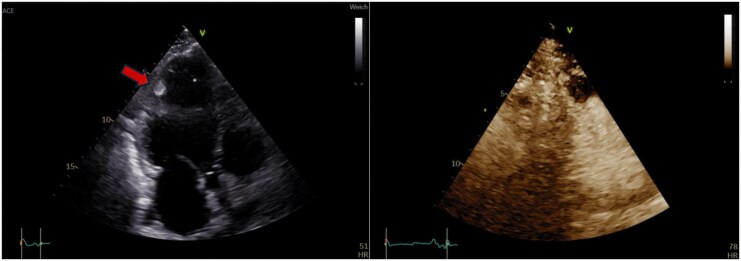
Contrast-enhanced echocardiography showing a mural left ventricular thrombus measuring 2 × 1.5 cm.

Cardiac magnetic resonance imaging (CMR) in June 2024 as undertaken to distinguish PPCM from alternative causes of non-ischaemic cardiomyopathy, including myocarditis and inherited cardiomyopathies. Cardiac magnetic resonance imaging demonstrated a dilated left ventricle with global hypokinesia, severe systolic dysfunction, patchy mid-wall late gadolinium enhancement (LGE) in the lateral and anterolateral segments, and a small pericardial effusion; no thrombus was visualized (*Figure 3*). Although compatible with non-ischaemic cardiomyopathy, the mid-wall LGE pattern was atypical for isolated PPCM, in which LGE is often absent or, if present, tends to be non-regional and patchy rather than segmental. This finding raised suspicion for an alternative or underlying cardiomyopathic process.^8^

**Figure 3:**
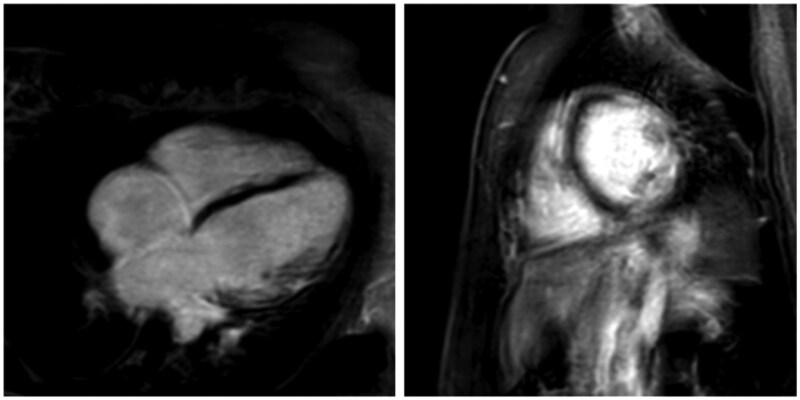
Cardiac magnetic resonance imaging demonstrating a markedly dilated, non-hypertrophied left ventricle with severely reduced systolic function and global hypokinesia. Patchy late gadolinium enhancement is observed in the mid-lateral, inferolateral, and anterolateral segments.

During outpatient follow-up, NT-proBNP gradually declined and LVEF improved modestly to 33%, with persistent global hypokinesia. Bromocriptine was discontinued in November 2024.

The combination of incomplete clinical recovery and the CMR fibrosis pattern prompted further diagnostic work-up, culminating in an endomyocardial biopsy in December 2024. Biopsy showed no acute or chronic lymphocytic myocarditis. Quantitative polymerase chain reaction detected parvovirus B19 deoxyribonucleic acid (DNA) at low viral load (342 copies/μg myocardial DNA) reflecting virus persistence without inflammatory infiltrates of myocarditis. Thus, the finding was considered incidental and did not alter management. Immunohistochemistry revealed a mosaic pattern in dystrophin staining in cardiomyocytes, characteristic of heterozygous dystrophin gene carriers (*Figure 4*).

**Figure 4:**
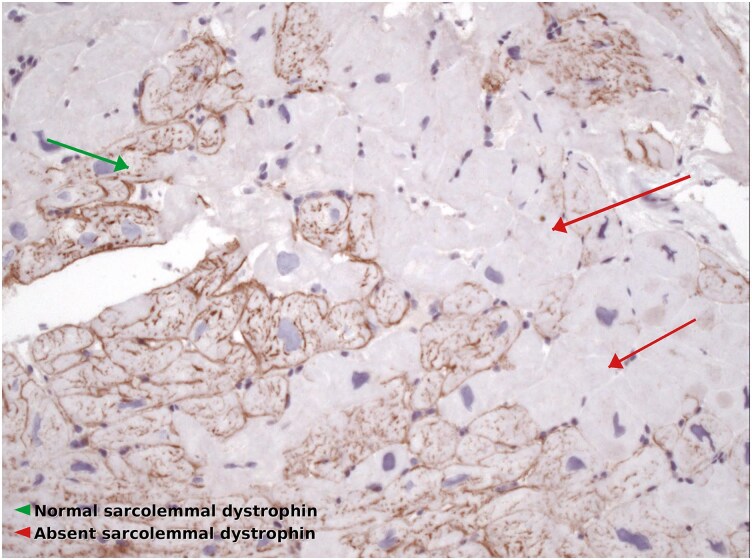
Immunohistochemical staining for dystrophin in endomyocardial biopsy shows preserved sarcolemmal expression in some cardiomyocytes (green arrow) and absent sarcolemmal dystrophin staining in others (red arrows), consistent with dystrophinopathy-associated cardiomyopathy.

Given the combined findings of persistent reduced LVEF, the CMR fibrosis pattern, and the mosaic pattern of dystrophin on biopsy, targeted genetic testing was undertaken in April 2025 in blood and identified a pathogenic X-linked deletion of exons 44–50 in the dystrophin gene, confirming X-linked dilated cardiomyopathy (XLDCM). DNA was extracted from whole blood and analysed by targeted panel sequencing on the Illumina® MiSeq™-system. A 223-gene panel covering channelopathy- and cardiomyopathy-associated genes was assessed by an accredited laboratory. This comprehensive, predefined cardiomyopathy and channelopathy panel was the laboratory’s standard diagnostic assay and includes all major genes associated with monogenic dilated cardiomyopathy, including titin; therefore, a narrower, disease-specific gene test for muscular dystrophy or dilated cardiomyopathy was not available within the routine clinical workflow. Variant analysis was performed according to guidelines of the American College of Medical Genetics and Genomics (ACMG) and cross-checked using established international databases and standard in silico prediction tools. The identified variant was classified as likely pathogenic according to ACMG criteria, based on PVS1 (predicted loss-of-function due to a multi-exon deletion in DMD, a gene in which loss-of-function is a well-established disease mechanism), PM2 (absence from population databases), and PP4 (a highly specific phenotype consistent with dystrophinopathy-associated cardiomyopathy, supported by mosaic dystrophin deficiency on endomyocardial biopsy and concordant cardiac magnetic resonance findings). This result unified the imaging and biopsy findings and explained the incomplete functional recovery. Although she reported no family history of cardiomyopathy, neuromuscular disease, or sudden unexplained death, it is noteworthy that her maternal grandfather had HF in his late 70s, received an implantable cardioverter-defibrillator (ICD), and died in 2022. The family was referred to specialized genetic counselling; cascade testing is ongoing.

In June 2025, due to persistently reduced ejection fraction, NYHA class II HF symptoms, and elevated arrhythmic risk, a subcutaneous ICD (S-ICD) was implanted. Repeat contrast echocardiography confirmed thrombus resolution, allowing discontinuation of anticoagulation. At follow-up, she remained stable (NYHA II) on GDMT.

## Discussion

This case demonstrates how atypical clinical or imaging features in presumed PPCM can reveal an underlying genetic cardiomyopathy, thereby necessitating a distinct diagnostic and management approach. Such diagnostic divergence aligns with the multifactorial pathophysiology of PPCM, which involves vascular dysfunction, hormonal derangements such as cleaved prolactin fragments, oxidative stress, and inflammation,^1–4^ all of which may impair myocardial function and unmask an underlying genetic predisposition.^9,10^

The initial presentation was compatible with PPCM. However, persistent systolic dysfunction, the atypical mid-wall LGE pattern, and mosaic loss of dystrophin prompted alternative diagnostic considerations. Low-level parvovirus B19 DNA in the absence of inflammation matched contemporary criteria for bystander presence rather than active viral infection. Identification of a pathogenic deletion in the dystrophin (DMD) gene established XLDCM as the underlying condition, with pregnancy serving as the physiological trigger. Female DMD carriers may develop isolated cardiomyopathy due to skewed X-chromosome inactivation, and haemodynamic stress during pregnancy may exceed myocardial reserve, precipitating overt disease.^9–12^

Important diagnostic principles emerge from this case. First, atypical imaging findings—particularly regional mid-wall fibrosis—should prompt consideration of inherited cardiomyopathy, for which early genetic testing may be appropriate within the initial cardiological work-up Second, the 2025 ESC Guidelines for the Management of Cardiovascular Disease in Pregnancy support genetic testing (Class IIa) when recovery is incomplete or when clinical features deviate from classical PPCM.^7^ Third, the genetic diagnosis refined the patient’s long-term prognosis and arrhythmic risk assessment and allowed targeted cascade screening of at-risk relatives, thereby informing both clinical management and family counselling. Genetic counselling and cascade genetic screening were initiated for the patient’s children and relatives, enabling early diagnostic follow-up and therapy and providing reassurance for those who did not carry the pathogenic variant.

Differential diagnostic considerations included viral myocarditis and idiopathic dilated cardiomyopathy. However, the absence of myocardial inflammation, mosaic dystrophin deficiency, and the proven DMD deletion confirmed XLDCM, with PPCM acting as the clinical trigger.^5,13–15^

Limitations include the single-patient nature of this observation and the unresolved penetrance among relatives. Although earlier genetic testing could have been considered in light of the atypical imaging findings, its turnaround time is inherently longer, whereas myocardial biopsy can deliver rapid diagnostic information when urgent evaluation is required. Nonetheless, its eventual implementation provided diagnostic clarity and enabled appropriate family screening.

In conclusion, PPCM may reveal unrecognized genetic cardiomyopathies. Early multimodal diagnostics and systematic genetic testing are essential for precision diagnosis and appropriate family management.

## Supplementary Material

ytag405_Supplementary_Data

## Data Availability

All relevant data are included within the manuscript and its Supplementary material.

## Lead author biography

Julian Bauer (born in Ahrweiler, Germany) is an assistant physician in the Department of Internal Medicine II, Cardiology, at Sana Klinikum Lichtenberg, Berlin, an academic teaching hospital of Charité – Universitätsmedizin Berlin. He studied medicine at Brandenburg Medical School and, prior to his medical studies, worked as a nurse in an intermediate care unit, where he developed an early interest in cardiology. His clinical interests focus on atrial fibrillation and cardiomyopathies.

## Notes

**Consent:** The authors confirm that written informed consent for submission and publication of this case report, including images and associated text, has been obtained from the patient in line with COPE guidance.
